# Supplementary material for: Evidence for spin current driven Bose-Einstein condensation of magnons
Source: Nat Commun. 2021 Nov 11;12:6541. doi: 10.1038/s41467-021-26790-y (PMC8585877; doi:10.1038/s41467-021-26790-y)
Supplement: Supplementary file 1 — Supplementary Information [file 41467_2021_26790_MOESM1_ESM.pdf]

Evidence for spin current driven Bose-Einstein condensation of magnons

Supplementary information

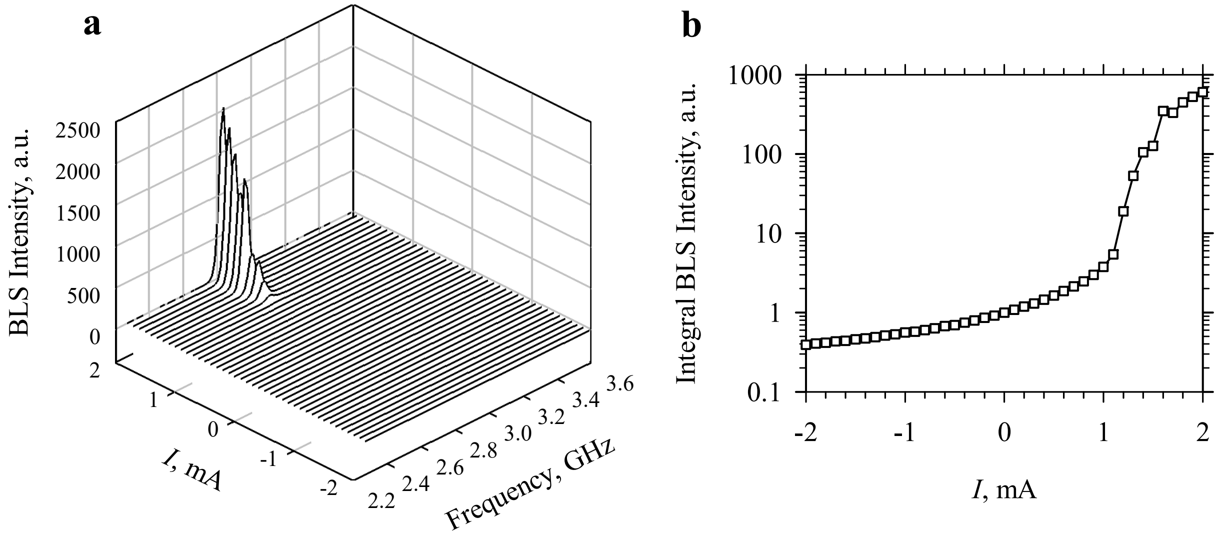

Supplementary Figure 1. Effects of the current polarity on the magnon population. a BLS spectra demonstrating the overpopulation of the lowest-energy magnon level. Note that the emergence of the BEC peak is observed for positive currents only, in agreement with the symmetry of the spin-Hall effect. **b** Current dependence of the integral BLS intensity. Note that the intensity monotonically decreases with the increase of the magnitude of the negative current. This fact clearly indicates that the contribution of the Spin Seebeck effect, which is symmetric with respect to the change of the current polarity, is negligible in the studied system. The data were obtained at $\mu_0 H_0$=100 mT.

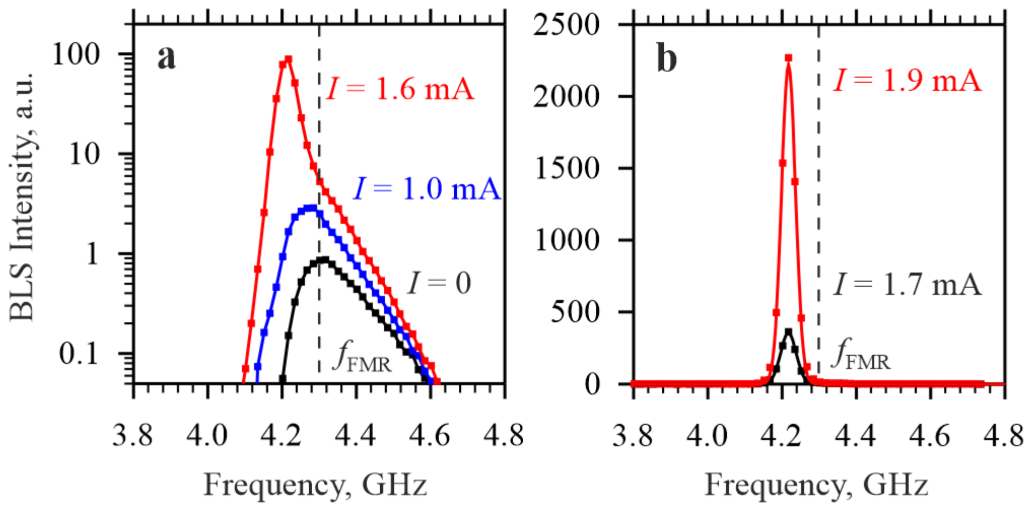

Supplementary Figure 2. Formation of the Bose-Einstein condensate of magnons. a and **b** Representative BLS spectra recorded at different currents in the Pt electrode, as labelled. Dashed lines mark the frequency of the ferromagnetic resonance. Note formation of the narrow intense spectral peak at I=1.7 mA. The data were obtained at $\mu_0 H_0$=150 mT.
